# Outcomes of Permanent Pacemaker Implantation in Patients with Pure Aortic Regurgitation after TAVI

**DOI:** 10.31083/RCM26543

**Published:** 2025-05-22

**Authors:** Changlin Ju, Yu Zhou, Tao Ge, Shengxin Tang, Zhigang Guo, Shiping Cao

**Affiliations:** ^1^Department of Cardiology, Nanfang Hospital, Southern Medical University, 510515 Guangzhou, Guangdong, China; ^2^Department of Cardiology, The First Affiliated Hospital of Wannan Medical College, 241000 Wuhu, Anhui, China; ^3^Department of Emergency, The First Affiliated Hospital of Wannan Medical College, 241000 Wuhu, Anhui, China

**Keywords:** transcatheter aortic valve implantation, pure aortic regurgitation, permanent pacemaker implantation, mortality, readmission rate, cardiac function

## Abstract

**Background:**

Transcatheter aortic valve implantation (TAVI) is increasingly utilized for patients with pure aortic regurgitation (PAR). A significant clinical challenge in this patient population is the need for permanent pacemaker implantation (PPI), which occurs frequently post-TAVI and can impact cardiac conduction and rhythm management. This study aimed to explore the effects of PPI on short-term mortality, rates of adverse events, and cardiac function in PAR patients following TAVI.

**Methods:**

This retrospective study, conducted in a single center, included 69 PAR patients who underwent TAVI from January 2021 to December 2023. Patients were categorized into two groups: those who received a permanent pacemaker (PM) and those who did not (NPM). The outcomes measured included complications such as pacemaker pocket hematoma and infection, changes in postoperative left ventricular ejection fraction (LVEF) and left ventricular end-diastolic diameter (LVEDD) at 6 months, as well as rates of rehospitalization and mortality.

**Results:**

No significant differences were noted in baseline characteristics or complications between the PM and NPM groups (*p* > 0.05). The types of PPI and associated complications were also comparable. There was no significant disparity in the incidence of all-cause mortality (PM: 12%, NPM: 11.36%, *p* = 0.755), major bleeding (PM: 4%, NPM: 4.55%, *p* = 0.612), or cerebral embolism (PM: 12%, NPM: 4.55%, *p* = 0.506) between the two groups at 6 months post-TAVI. Additionally, readmission rates were similar at 1, 3, and 6 months following the procedure. Multinomial logistic regression analysis revealed that age (*p* = 0.020), history of cerebral infarction (*p* = 0.015), and hypertension (*p* = 0.019) were significant predictors of mortality. The survival curve indicated that fatalities in the NPM group predominantly occurred during the perioperative period. At the 6-month follow-up, there was no significant difference in survival rates between the two groups (*p* = 0.971). Regarding cardiac function, irrespective of PPI, a decreasing trend in LVEDD (PM: –4.19 mm, NPM: –6.16 mm, *p* = 0.000) and an increasing trend in LVEF (PM: +2.19%, NPM: +2.74%, *p* = 0.053) were observed.

**Conclusions:**

This study was the first to investigate the effects of PPI on the short-term mortality, adverse events, and cardiac function of PAR after TAVI. The results indicated that for PAR, advanced age and previous cerebral embolism increase the mortality after TAVI; however, PPI was not associated with mortality and adverse events after 6 months.

## 1. Introduction

Transcatheter aortic valve implantation (TAVI) is predominantly utilized for patients diagnosed with aortic stenosis (AS). However, with the ongoing advancements in valve technology and stent design, a growing cohort of patients with pure aortic regurgitation (PAR) are receiving TAVI [1]. A common complication associated with TAVI is the need for permanent pacemaker implantation (PPI). The incidence of PPI following atrioventricular block in AS patients undergoing TAVI varies between 3.4% and 25.9% [2]. In contrast to AS, PAR, characterized by regurgitated flow due to aortic valve leaflet dysfunction, leads to left ventricular dilation and volume overload, presenting unique challenges compared to the calcific stenosis in AS. This distinction may impact TAVI procedures and the need for PPI, with PAR patients showing a higher PPI requirement post-TAVI, likely due to the altered left ventricular outflow tract geometry and increased risk of conduction disturbances [3].

Although prior research has indicated that pacemaker implantation, particularly with leads positioned in the right ventricular apex, is associated with an increased risk of heart failure and atrial fibrillation [4, 5], the impact of PPI on cardiac function following TAVI in AS patients remains a subject of debate [6]. A growing body of clinical trials suggests that PPI may increase hospitalization rates and mortality among these individuals [7, 8]. Consequently, this study is designed to investigate the effects of PPI on PAR patients during a six-month follow-up period after TAVI, addressing a gap in the literature where the influence of PPI on cardiac function and prognosis in PAR patients post-TAVI has yet to be documented.

By bridging this knowledge gap, this research will provide valuable insights into the effects of PPI on cardiac function and prognosis, which will be instrumental in refining patient selection, procedural strategies, and post-procedure management. Ultimately, these findings will contribute to the development of guidelines for the management of PAR patients undergoing TAVI, with a particular focus on the decision-making process for PPI.

## 2. Materials and Methods

### 2.1 Study Population

This single-center, retrospective, and consecutive study encompassed all PAR patients who underwent TAVI at our institution from January 2021 to December 2023. Patients who successfully received TAVI were categorized into two groups based on the necessity for PPI post-TAVI: the permanent pacemaker (PM) group and the group who did not receive a permanent pacemaker (NPM). The study recorded the incidence of complications such as pacemaker pocket hematoma, infection, changes in postoperative cardiac function at six months, rehospitalization rates, and mortality rates. Inclusion criteria for PAR patients included symptomatic severe PAR and a Society of Thoracic Surgeons (STS) risk score of ≥4%, indicating a high surgical risk. Exclusion criteria: (1) Left ventricular thrombus; (2) Left ventricular outflow obstruction; (3) Anatomical unsuitability for TAVI (e.g., high risk of coronary artery occlusion); (4) Contraindications for anticoagulation; (5) A life expectancy of less than 12-month post-correction of valve disease; (6) Prior pacemaker implantation before TAVI; (7) TAVI failure. This study was conducted in accordance with the ethical standards of the 1964 Declaration of Helsinki and its later amendments. All procedures involving human participants were approved by the hospital’s Ethics Committee, and informed consent was obtained from all participants.

### 2.2 TAVI Procedure

The TAVI procedure was performed utilizing the VitaFlow Liberty system via the femoral artery. The size of the prosthesis was determined based on a computed tomography scan of the aortic ring area. Aortic valve positioning was guided by angiography and trans esophageal echocardiography. The valve was deployed at the level of the coronary sinus under rapid pacing (≥160 beats/min). Post-procedure, patients received standard care management, which included transthoracic echocardiography and electrocardiogram monitoring at discharge.

### 2.3 PPI Procedure

All patients underwent temporary pacemaker implantation via the right internal jugular vein prior to TAVI. PPI was performed if severe bradycardia persisted for 5 to 7 days post-TAVI without resolution. The right ventricular lead was positioned in the mid to lower septum. Cardiac resynchronization therapy (CRT) was administered to patients with a left ventricular ejection fraction (LVEF) of <50% and ventricular pacing dependence, in strict accordance with pacemaker implantation guidelines [9]. All procedures were conducted by experienced interventional cardiologists following established care protocols.

### 2.4 Collection and Definition of Covariates

(1) Hypertension is a major risk factor for cardiovascular events and is closely monitored in patients undergoing TAVI [10, 11].

(2) Diabetes mellitus is associated with increased morbidity and mortality in cardiovascular patients, including those undergoing valve interventions [12].

(3) Coronary artery disease confirmed by coronary angiography or computed tomography angiography with coronary artery stenosis of 50% or greater. The presence of coronary artery disease can complicate outcomes following TAVI and is an important comorbidity to consider [13].

(4) Atrial fibrillation is a common arrhythmia that can affect patient management and outcomes post-TAVI [14].

(5) Mitral regurgitation was diagnosed via doppler echocardiography, mitral regurgitation can significantly impact left ventricular function and is a relevant comorbidity in patients with aortic valve disease [15].

(6) The incidence of hematoma and major bleeding in TAVI patients following pacemaker implantation was observed. Major bleeding was defined by the occurrence of one of the following three conditions: fatal bleeding; symptomatic bleeding in critical locations or organs, such as intracranial, spinal, intraocular, peritoneal, intra-articular, pericardial, or intramuscular compartment syndrome; or a decrease in hemoglobin by 20 g/L (1.2 mmol/L) or more, resulting in the transfusion of two or more units of whole blood or red blood cells [16].

### 2.5 Statistical Analysis

**Fig. 1.**
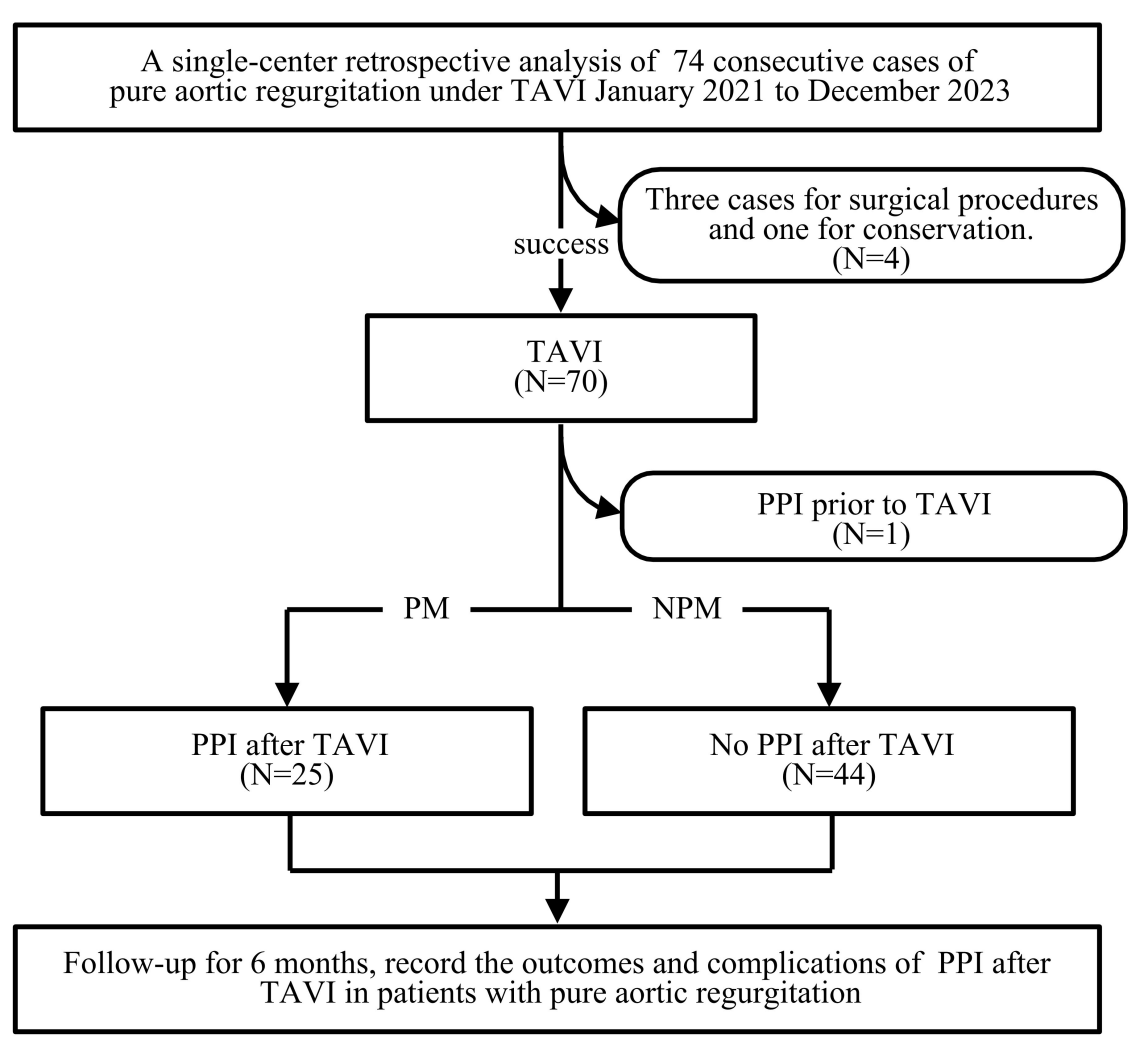
**Study flow diagram**. TAVI, transcatheter aortic valve implantation; PPI, permanent pacemaker implantation; PM, permanent pacemaker; NPM, no permanent pacemaker.

Data were analyzed using SPSS Statistics 26.0 (IBM, Armonk, NY, USA). Chi-square and Fisher’s exact tests were employed to evaluate associations between outcomes and categorical variables. The *t*-test was utilized to compare means of continuous variables between patient groups and the Mann-Whitney U test was used for abnormally distributed data. Repeated measures ANOVA of PPI on left ventricular end-diastolic diameter (LVEDD) and LVEF after TAVI, and stepwise regression analysis of risk factors for mortality after TAVI on PAR patients was conducted to identify factors associated with six-month mortality, utilizing hazard ratios. The Kaplan-Meier survival curve, along with the log-rank test, was employed to compare six-month mortality, with *p* < 0.05 indicating statistical significance (Fig. 1).

## 3. Results

### 3.1 Baseline Data

In this study, 74 patients with PAR underwent TAVI. After excluding four cases of failure and one patient with a previously implanted permanent pacemaker, 69 patients with a successfully implanted TAVI were included. Among these, 25 patients required postoperative implantation of a permanent pacemaker and were classified into the PM group, while 44 patients did not require a pacemaker and were classified into the NPM group. No significant differences in baseline characteristics or complications were observed between the two groups (*p* > 0.05) (Table 1).

**Table 1.** **Baseline characteristics in patient with TAVI**.

Parameters	NPM (n = 44)	PM (n = 25)	*p*-value
Age (Y)	73.55 ± 8.02	73.64 ± 7.34	0.961
Gender (male, %)	27 (61.36)	14 (56.00)	0.663
BMI (kg/m^2^)	23.08 ± 2.23	22.96 ± 2.03	0.832
Creatinine (µmol/L)	111.57 ± 96.99	98.91 ± 87.55	0.592
Uric acid (mmol/L)	315.82 ± 189.06	344.93 ± 184.15	0.537
Glucose (mmol/L)	4.83 ± 1.28	5.25 ± 1.15	0.179
Cholesterol (mmol/L)	3.46 ± 1.10	3.45 ± 1.63	0.987
Triglyceride (mmol/L)	0.98 ± 0.55	1.10 ± 0.87	0.517
HDL-c (mmol/L)	1.29 ± 0.39	1.26 ± 0.51	0.758
LDL-c (mmol/L)	1.80 ± 0.90	1.86 ± 1.06	0.787
Lipoprotein(a) (mmol/L)	140.40 (19.02, 341.10)	87.00 (13.70, 226.70)	0.427
Prothrombin time (S)	14.09 ± 5.97	12.74 ± 1.27	0.156
INR	1.23 ± 0.54	1.10 ± 0.11	0.129
APTT (S)	31.75 ± 14.82	35.32 ± 22.13	0.430
Platelet count (10^9^/L)	134.43 ± 63.09	134.20 ± 47.03	0.987
Prosthesis size (mm)	28.61 ± 1.75	28.44 ± 2.14	0.717
NYHA association	3.18 ± 0.79	3.00 ± 0.58	0.316
Hypertension (N, %)	25 (56.82)	11 (44.00)	0.306
Diabetes mellitus (N, %)	5 (11.36)	2 (8.00)	0.976
Cerebral embolism (N, %)	5 (11.36)	1 (4.00)	0.297
CAD (N, %)	12 (27.91)	5 (20.00)	0.468
Atrial fibrillation (N, %)	16 (36.36)	6 (24.00)	0.289
Mitral regurgitation (N, %)	6 (13.64)	5 (20.00)	0.632

Y, year; BMI, body mass index; HDL-c, high-density lipoprotein cholesterol; LDL-c, low-density lipoprotein cholesterol; CAD, coronary artery disease; NYHA, New York Heart Association; APTT, activated partial thromboplastin time; INR, international normalized ratio; *p* < 0.05 indicates a statistically significant difference.

### 3.2 Types of PPI and Complications

25 patients received single-chamber, dual-chamber, or CRT pacemakers. One patient was treated with warfarin, three with rivaroxaban, fourteen with aspirin plus clopidogrel, one with aspirin plus clopidogrel plus rivaroxaban, and five with aspirin plus clopidogrel plus low molecular weight heparin. There were no significant differences in various anticoagulation regimens between the two groups (*p* > 0.05). During the follow-up period, no incidents of pocket hematoma, infection, or cardiac perforation were reported among all PPI patients (Table 2).

**Table 2.** **Antithrombotic therapy regimen and complications following TAVI for PAR patients**.

Parameters		PM (N = 25)	NPM (N = 44)	*p*-value
Warfarin (N, %)		1 (4.00)	2 (4.55)	0.612
Rivaroxaban (N, %)		3 (12.00)	5 (11.36)	0.755
Asprin+Clopidogrel (N, %)		14 (56.00)	20 (45.45)	0.400
Clopidogrel+Heparin (N, %)		1 (4.00)	4 (9.09)	0.763
Asprin+Clopidogrel+Rivaroxaban (N, %)		1 (4.00)	3 (6.82)	0.957
Asprin+Clopidogrel+Heparin (N, %)		5 (20.00)	10 (22.73)	0.792
Pacemaker				
	VVI (N, %)	5 (20.00)	-	-
	DDD (N, %)	19 (76.00)	-	-
	CRT (N, %)	1 (4.00)	-	-
Complications				
	Pocket hematoma	0	-	-
	Pocket infection	0	-	-
	Electrode displacement	0	-	-

PAR, pure aortic regurgitation; VVI, ventricular demand pacing; DDD, dual-chamber pacing; CRT, cardiac resynchronization therapy. *p* < 0.05 indicates a statistically significant difference.

### 3.3 Effects of PPI on LVEDD and LVEF after TAVI

Patients with PAR who underwent TAVI were followed up for six months. Excluding the eight deceased patients, the remaining 61 were analyzed for changes in baseline echocardiographic parameters, LVEDD and LVEF, at six-month follow-up, and the impact of PPI on these parameters was assessed. Repeated measures ANOVA for changes in LVEDD revealed significant main effects of follow-up time in both PM and NPM groups (*p* = 0.000), indicating that the LVEDD was significantly reduced after TAVI regardless of PPI (Table 3). LVEF exhibited an upward trend, but repeated measures ANOVA for changes in LVEF revealed no significant main effects of follow-up time in both the PM and NPM groups (*p* = 0.053), which was also seen in the group effect (*p* = 0.652) and the interaction effect between group and follow-up time (*p* = 0.789). This suggests that regardless of PPI, although there was an increase in LVEF during the follow-up period, there were no significant differences between or within the groups (Table 3).

**Table 3.** **Repeated measures ANOVA of PPI on LVEDD and LVEF after TAVI**.

Parameters	LVEDD (mm)		LVEF (%)	
	PM (N = 20)	NPM (N = 41)	PM (N = 20)	NPM (N = 41)
Baseline	56.50 ± 7.33	57.23 ± 6.24	55.81 ± 9.91	54.84 ± 9.76
3 month follow-up	52.07 ± 7.19	53.16 ± 6.54	56.69 ± 8.31	54.81 ± 9.44
6 month follow-up	52.31 ± 6.58	51.07 ± 5.43	58.00 ± 6.18	57.58 ± 7.81
Group *p* value	0.917		0.652	
Time *p* value	0.000*		0.053	
Group by time *p*-value	0.158		0.789	

LVEDD, left ventricular end-diastolic diameter; LVEF, left ventricular ejection fraction; Data are presented as mean ± SE, * *p* < 0.05 indicates a statistically significant difference.

### 3.4 Adverse Events after TAVI

No significant differences in the incidence of all-cause mortality, major bleeding, or cerebral embolism were observed between the PM and NPM groups six months post-TAVI (*p* > 0.05). Additionally, no significant differences in readmission rates were noted between the two groups at one month, three months, or six months post-TAVI (*p* > 0.05) (Table 4).

**Table 4.** **Adverse events during the 6-month follow-up after TAVI for PAR patients**.

Parameters		PM (N = 25)	NPM (N = 44)	χ ^2^	*p*-value
Adverse events		13 (59.09)	18 (43.18)	1.486	0.223
Major bleeding		1 (4.0)	2 (4.55)	0.257	0.612Δ
Cerebral embolism		3 (12)	2 (4.55)	0.442	0.506
All-cause mortality		3 (12)	5 (11.36)	0.097	0.755
Follow-up					
	one-month readmission	2 (8)	3 (6.82)	0.091	0.763
	three-month readmission	4 (16)	8 (18.18)	0.010	0.920
	six-month readmission	6 (24)	10 (22.73)	0.014	0.904

Δ Fisher’s chi-square test, the rest use Pearson’s chi-square test.

### 3.5 Risk Factors for Mortality

As depicted in Table 5, a stepwise regression analysis was conducted for all variables, including patient basic parameters and key observation indicators such as LVEF, pacemaker implantation, and other variables within the model, with death as the dependent variable. The findings indicated that age, serum creatinine, and cerebral infarction significantly increased the risk of mortality, with each unit increase in cerebral infarction leading to a 47.718-fold increase in the incidence of death. In contrast, hypertension had a significantly negative impact on mortality (*p* = 0.019), whereas pacemaker implantation and LVEF were not statistically significant (*p* > 0.05).

**Table 5.** **Stepwise Regression Analysis of risk factors for mortality after TAVI on PAR patients**.

Parameters	Stepwise regression analysis			
	coefficient	*p*-value	odds ratio	95% CI
Age	0.238	0.020	1.268	1.038–1.550
Creatinine	0.010	0.032	1.010	1.001–1.019
Cerebral embolism	3.865	0.015	47.718	2.091–1088.739
Hypertension	–3.621	0.019	0.027	0.001–0.552

McFadden R-squared = 0.378, Cox & Snell R-squared = 0.239, Nagelkerke R-squared = 0.46.

### 3.6 Kaplan-Meier Survival Analysis

**Fig. 2.**
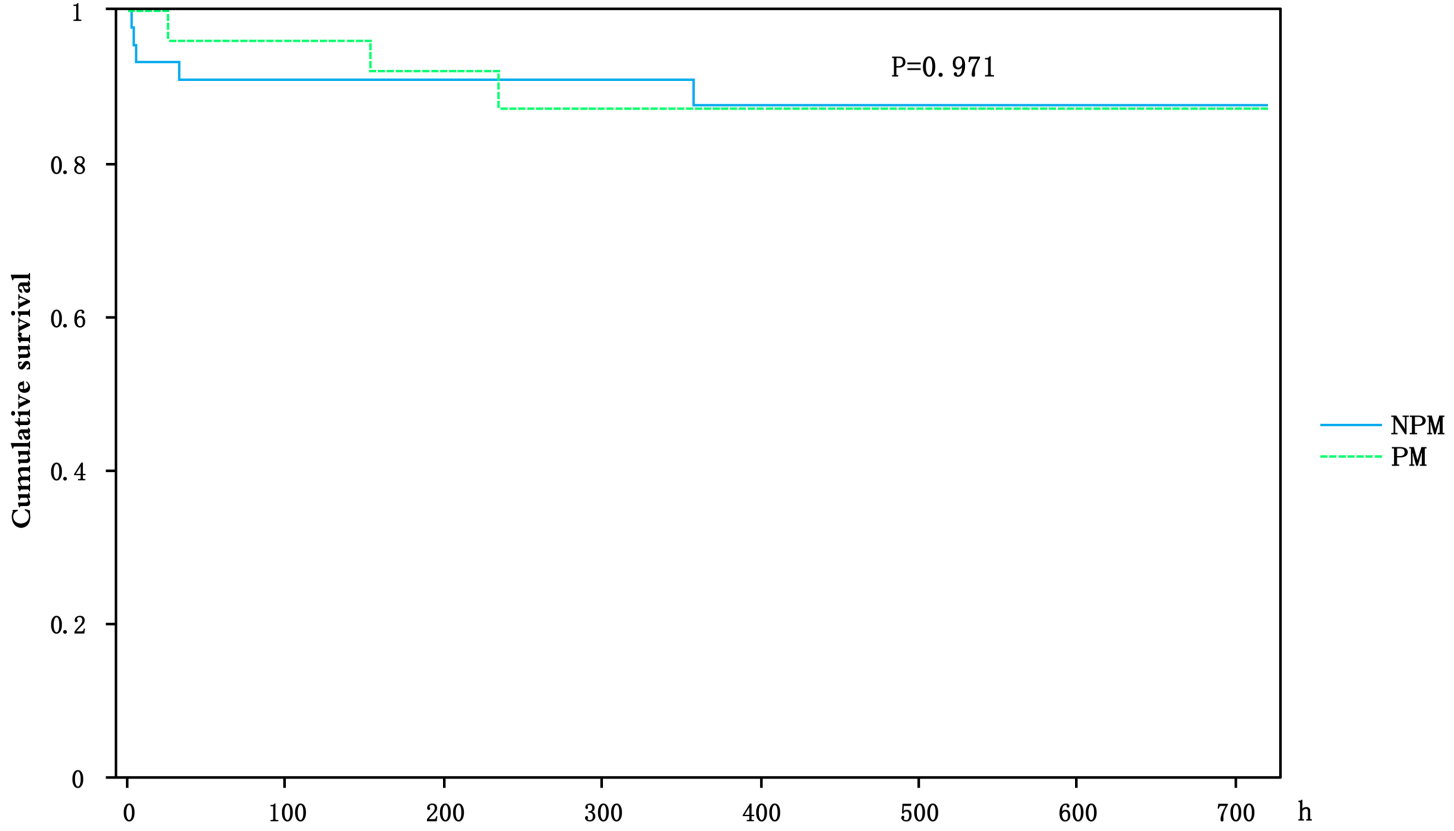
**The impact of PPI or not on survival among pure aortic regurgitation patients after TAVI**.

The survival curve indicated that mortality among NPM patients predominantly occurred during the perioperative period. No significant differences in survival rates were observed between the two groups at six months post-TAVI (*p* = 0.971) (Fig. 2).

## 4. Discussion

This study represents the first investigation into the effects of PPI on short-term mortality, adverse events, and cardiac function in PAR patients following TAVI. The findings indicate that PPI is not associated with mortality or the incidence of adverse events after six months among PAR patients. Factors such as advanced age, elevated creatinine levels, and prior cerebral embolism were identified as contributors to increased mortality post-TAVI.

Regarding the impact of PPI on cardiac structure and function post-TAVI, the PACE-TAVI registry revealed that AS patients with a right ventricular pacing (RVP) ratio of <40% exhibited improved cardiac function post-TAVI compared to preoperative levels; however, those with an RVP ratio >40% experienced diminished cardiac function and a higher rate of heart failure-related rehospitalization [7]. A recent meta-analysis indicated that RVP is associated with a 2.9% reduction in LVEF, alongside reductions in left ventricular stroke volume and increases in both left ventricular end-diastolic and end-systolic diameters [17, 18]. This is consistent with previous literature, which states that long-term right ventricular pacing has been associated with right ventricular desynchronization, negative left ventricular remodeling, and heart failure. In contrast, physiological pacing methods, such as His-bundle pacing and left bundle branch area pacing (LBBaP), have emerged as novel physiological pacing modalities, showing excellent results for patients with conventional indications for bradycardia pacing [19]. In Wang *et al*.’s study [20], patients who underwent TAVI and received either RVP or LBBaP exhibited a significant reduction in LVEDD over a five-year follow-up period, irrespective of their baseline LVEF being below 50%. Additionally, both groups demonstrated a notable enhancement in LVEF, with the LBBaP group showing a more marked improvement [20]. This study demonstrated that among AR patients post-TAVI, regardless of PPI, a trend towards decreased left ventricular size and increased LVEF was observed. Notably, significant alterations in left ventricular dimensions and LVEF may manifest with extended follow-up.

The relationship between PPI and mortality or rehospitalization rates post-TAVI remains contentious. Numerous studies have established that PPI correlates with increased rehospitalization and mortality rates among patients undergoing TAVI [21, 22]. The PACE-TAVI registry also indicated that patients with an RVP ratio >40% faced heightened cardiovascular mortality and heart failure rehospitalization rates [7]. A four-year follow-up study confirmed that PPI can elevate heart failure hospitalization rates and adversely affect cardiac function recovery, particularly in patients with prior LVEF <50% [8]. In patients with LVEF ≤40%, CRT was associated with improved survival compared to non-CRT [23]. For TAVI patients with preserved LVEF, postoperative left ventricular desynchronization due to high-burden RVP or permanent left bundle branch block was linked to a significantly increased risk of death and cardiomyopathy at one-year follow-up [24, 25]. A five-year follow-up after TAVI revealed that patients with RVP had a significantly higher risk of readmission compared to those with LBBaP (21.4% vs. 7.5%; 95% CI: 1.01 to 5.08; *p* = 0.048) [20]. However, Mohananey *et al*. [26] analyzed outcomes in patients with PPI and without PPI after TAVI at 30-day and one-year follow-ups, finding no significant differences in all-cause mortality, cardiovascular mortality, or myocardial events between the groups. In this study, PPI were not associated with rehospitalization or mortality, possible reasons include the short follow-up period of this study, where the short-term impact of PPI on cardiac function may not be significant, but long-term effects could lead to increased hospitalization for heart failure and mortality; the small sample size and lack of stratification based on patients’ ejection fraction (EF) values may also have influenced the results of this experiment, necessitating further research to elucidate the prognostic implications of varying LVEF levels among PAR patients undergoing TAVI.

The debate surrounding whether PPI increases mortality post-TAVI persists, yet other factors contributing to elevated mortality rates have been identified. Advanced age and myocardial fibrosis have been shown to increase mortality rates among AS patients post-TAVI [27]. In a cohort of 500 patients undergoing TAVR, with a median follow-up of 5.2 years, advanced age, male gender, chronic kidney disease stage ≥3, diabetes mellitus, and coronary heart disease were associated with heightened mortality risk, while coronary artery bypass grafting did not mitigate this risk [28]. Interestingly, hypertension did not significantly impact mortality [26]. Post-TAVI bleeding, particularly major or life-threatening bleeding, was found to elevate the 30-day postoperative mortality of rates [29]. A large meta-analysis by Eggebrecht *et al*. [30] reported that the 30-day mortality rate following a stroke was 3.5 times higher. The one-month mortality rate reached 25% among patients with cerebral embolism, compared to 7% for those without [31]. This study corroborated that advanced age and cerebral infarction increased mortality rates post-TAVI, while hypertension unexpectedly emerged as a protective factor, potentially due to systemic hypotension leading to cerebral hypo perfusion, thereby increasing mortality risk [31].

## 5. Limitations

The small sample size and the short follow-up period constrain the generalizability of our findings and limit our capacity to assess the long-term outcomes effectively. Future research endeavors should aim to incorporate larger cohorts and extended follow-up periods to more accurately determine the effects of PPI on survival and cardiac function in PAR patients post-TAVI. Furthermore, the absence of LVEF stratification in our analysis represents a significant limitation, suggesting a clear need for future studies to explore the nuanced impact of PPI across different LVEF levels. Such larger, longitudinal studies are essential to establish the definitive role of PPI in the management of PAR patients following TAVI, ultimately informing clinical practice and patient care standards.

## 6. Conclusions

Our study, the first to examine the short-term impact of PPI on patients with PAR following TAVI, reveals no association between PPI and increased mortality or adverse events within the initial six-month follow-up. Notably, advanced age, elevated creatinine levels, and a history of cerebral infarction emerged as significant predictors of mortality, thereby underscoring their critical role in post-TAVI care. Overall, these findings provide valuable insights into the management strategies for PAR patients in the aftermath of TAVI, highlighting the need for tailored approaches based on individual patient characteristics.

## Data Availability

The datasets used and/or analyzed during the current study are available from the corresponding author on reasonable request.

## Abbreviations

AS, aortic stenosis; CRT, cardiac resynchronization therapy; LBBaP, left bundle branch area pacing; LVEDD, left ventricular end-diastolic diameter; LVEF, left ventricular ejection fraction; PAR, pure aortic regurgitation; PPI, permanent pacemaker implantation; RVP, right ventricular pacing; TAVI, transcatheter aortic valve implantation.

## References

[1] Ullah W, Suleiman ARM, Osman H, Bodempudi S, Muhammadzai HZU, Zahid S (2024). Trends and Outcomes of Transcatheter Aortic Valve Implantation in Aortic Insufficiency: A Nationwide Readmission Database Analysis. *Current Problems in Cardiology*.

[2] Szotek M, Drużbicki Ł, Sabatowski K, Amoroso GR, De Schouwer K, Matusik PT (2023). Transcatheter Aortic Valve Implantation and Cardiac Conduction Abnormalities: Prevalence, Risk Factors and Management. *Journal of Clinical Medicine*.

[3] Zhang X, Liang C, Zha L, Zuo Q, Hu G, Ding J (2024). Predictors for new-onset conduction block in patients with pure native aortic regurgitation after transcatheter aortic valve replacement with a new-generation self-expanding valve (VitaFlow Liberty™): a retrospective cohort study. *BMC Cardiovascular Disorders*.

[4] Osiecki A, Kochman W, Witte KK, Mańczak M, Olszewski R, Michałkiewicz D (2022). Cardiomyopathy Associated with Right Ventricular Apical Pacing-Systematic Review and Meta-Analysis. *Journal of Clinical Medicine*.

[5] Cicchitti V, Radico F, Bianco F, Gallina S, Tonti G, De Caterina R (2016). Heart failure due to right ventricular apical pacing: the importance of flow patterns. *Europace: European Pacing, Arrhythmias, and Cardiac Electrophysiology: Journal of the Working Groups on Cardiac Pacing, Arrhythmias, and Cardiac Cellular Electrophysiology of the European Society of Cardiology*.

[6] Sammour Y, Krishnaswamy A, Kumar A, Puri R, Tarakji KG, Bazarbashi N (2021). Incidence, Predictors, and Implications of Permanent Pacemaker Requirement After Transcatheter Aortic Valve Replacement. *JACC. Cardiovascular Interventions*.

[7] Bruno F, Munoz Pousa I, Saia F, Vaira MP, Baldi E, Leone PP (2023). Impact of Right Ventricular Pacing in Patients With TAVR Undergoing Permanent Pacemaker Implantation. *JACC. Cardiovascular Interventions*.

[8] Chamandi C, Barbanti M, Munoz-Garcia A, Latib A, Nombela-Franco L, Gutiérrez-Ibanez E (2018). Long-Term Outcomes in Patients With New Permanent Pacemaker Implantation Following Transcatheter Aortic Valve Replacement. *JACC. Cardiovascular Interventions*.

[9] Glikson M, Nielsen JC, Kronborg MB, Michowitz Y, Auricchio A, Barbash IM (2021). 2021 ESC Guidelines on cardiac pacing and cardiac resynchronization therapy [published erratum in European Heart Journal. 2022; 43: 1651. https://doi.org/10.1093/eurheartj/ehac075]. *European Heart Journal*.

[10] Whelton PK, Carey RM, Aronow WS, Casey DE, Collins KJ, Dennison Himmelfarb C (2018). 2017 ACC/AHA/AAPA/ABC/ACPM/AGS/APhA/ASH/ASPC/NMA/PCNA Guideline for the Prevention, Detection, Evaluation, and Management of High Blood Pressure in Adults: A Report of the American College of Cardiology/American Heart Association Task Force on Clinical Practice Guidelines. *Hypertension (Dallas, Tex.: 1979)*.

[11] Perlman GY, Loncar S, Pollak A, Gilon D, Alcalai R, Planer D (2013). Post-procedural hypertension following transcatheter aortic valve implantation: incidence and clinical significance. *JACC. Cardiovascular Interventions*.

[12] van Nieuwkerk AC, Santos RB, Mata RB, Tchétché D, de Brito FS, Barbanti M (2022). Diabetes mellitus in transfemoral transcatheter aortic valve implantation: a propensity matched analysis. *Cardiovascular Diabetology*.

[13] Puri R (2024). The Art and Science of Managing Stable Coronary Artery Disease in Patients Undergoing TAVI. *The New England Journal of Medicine*.

[14] Van Mieghem NM, Unverdorben M, Hengstenberg C, Möllmann H, Mehran R, López-Otero D (2021). Edoxaban versus Vitamin K Antagonist for Atrial Fibrillation after TAVR. *The New England Journal of Medicine*.

[15] Muratori M, Fusini L, Tamborini G, Ghulam Ali S, Gripari P, Fabbiocchi F (2020). Mitral valve regurgitation in patients undergoing TAVI: Impact of severity and etiology on clinical outcome. *International Journal of Cardiology*.

[16] Schulman S, Kearon C, Subcommittee on Control of Anticoagulation of the Scientific and Standardization Committee of the International Society on Thrombosis and Haemostasi (2005). Definition of major bleeding in clinical investigations of antihemostatic medicinal products in non-surgical patients. *Journal of Thrombosis and Haemostasis: JTH*.

[17] Faroux L, Chen S, Muntané-Carol G, Regueiro A, Philippon F, Sondergaard L (2020). Clinical impact of conduction disturbances in transcatheter aortic valve replacement recipients: a systematic review and meta-analysis. *European Heart Journal*.

[18] Yakubov SJ, Amin A (2023). Pacing After TAVR: Just Give Me the Beat, But Improve the Instruments. *JACC. Cardiovascular Interventions*.

[19] Zhou Y, Wang J, Wei Y, Zhang W, Yang Y, Rui S (2022). Left ventricular septal pacing versus left bundle branch pacing in the treatment of atrioventricular block. *Annals of Noninvasive Electrocardiology: the Official Journal of the International Society for Holter and Noninvasive Electrocardiology, Inc*.

[20] Wang X, Xu Y, Zeng L, Tan K, Zhang X, Han X (2024). Long-term outcomes of left bundle branch area pacing compared with right ventricular pacing in TAVI patients. *Heart Rhythm*.

[21] Zito A, Princi G, Lombardi M, D’Amario D, Vergallo R, Aurigemma C (2022). Long-term clinical impact of permanent pacemaker implantation in patients undergoing transcatheter aortic valve implantation: a systematic review and meta-analysis. *Europace: European Pacing, Arrhythmias, and Cardiac Electrophysiology: Journal of the Working Groups on Cardiac Pacing, Arrhythmias, and Cardiac Cellular Electrophysiology of the European Society of Cardiology*.

[22] Auffret V, Boulmier D, Didier R, Leurent G, Bedossa M, Tomasi J (2024). Clinical effects of permanent pacemaker implantation after transcatheter aortic valve implantation: Insights from the nationwide FRANCE-TAVI registry. *Archives of Cardiovascular Diseases*.

[23] Kirchner J, Gerçek M, Sciacca V, Reil JC, Guckel D, Potratz M (2024). Mortality after cardiac resynchronization therapy or right ventricular pacing in transcatheter aortic valve replacement recipients. *Clinical Research in Cardiology: Official Journal of the German Cardiac Society*.

[24] Ananwattanasuk T, Atreya AR, Teerawongsakul P, Ghannam M, Lathkar-Pradhan S, Latchamsetty R (2023). Outcomes in patients with electrocardiographic left ventricular dyssynchrony following transcatheter aortic valve replacement. *Heart Rhythm*.

[25] Fadahunsi OO, Olowoyeye A, Ukaigwe A, Li Z, Vora AN, Vemulapalli S (2016). Incidence, Predictors, and Outcomes of Permanent Pacemaker Implantation Following Transcatheter Aortic Valve Replacement: Analysis From the U.S. Society of Thoracic Surgeons/American College of Cardiology TVT Registry. *JACC. Cardiovascular Interventions*.

[26] Mohananey D, Jobanputra Y, Kumar A, Krishnaswamy A, Mick S, White JM (2017). Response by Mohananey et al to Letter Regarding Article, “Clinical and Echocardiographic Outcomes Following Permanent Pacemaker Implantation After Transcatheter Aortic Valve Replacement: Meta-Analysis and Meta-Regression”. *Circulation. Cardiovascular Interventions*.

[27] Alnour F, Beuthner BE, Hakroush S, Topci R, Vogelgesang A, Lange T (2024). Cardiac fibrosis as a predictor for sudden cardiac death after transcatheter aortic valve implantation. *EuroIntervention: Journal of EuroPCR in Collaboration with the Working Group on Interventional Cardiology of the European Society of Cardiology*.

[28] Thogata H, Garikipati S, Reddy S S, Abhinav Reddy P, Kumar Jella H (2023). Long-Term Prognosis and Predictors of Mortality in Patients Undergoing Transcatheter Aortic Valve Replacement: A Retrospective Analysis. *Cureus*.

[29] Wang J, Yu W, Jin Q, Li Y, Liu N, Hou X (2017). Risk Factors for Post-TAVI Bleeding According to the VARC-2 Bleeding Definition and Effect of the Bleeding on Short-Term Mortality: A Meta-analysis. *The Canadian Journal of Cardiology*.

[30] Eggebrecht H, Schmermund A, Voigtländer T, Kahlert P, Erbel R, Mehta RH (2012). Risk of stroke after transcatheter aortic valve implantation (TAVI): a meta-analysis of 10,037 published patients. *EuroIntervention: Journal of EuroPCR in Collaboration with the Working Group on Interventional Cardiology of the European Society of Cardiology*.

[31] Armijo G, Nombela-Franco L, Tirado-Conte G (2018). Cerebrovascular Events After Transcatheter Aortic Valve Implantation. *Frontiers in Cardiovascular Medicine*.

